# ZnO Thin Films Growth Optimization for Piezoelectric Application

**DOI:** 10.3390/s21186114

**Published:** 2021-09-12

**Authors:** Vincent Polewczyk, Riccardo Magrin Maffei, Giovanni Vinai, Matteo Lo Cicero, Stefano Prato, Pietro Capaldo, Simone Dal Zilio, Alessandro di Bona, Guido Paolicelli, Andrea Mescola, Sergio D’Addato, Piero Torelli, Stefania Benedetti

**Affiliations:** 1 Laboratorio TASC, Istituto Officina dei Materiali (IOM)-CNR, 34149 Trieste, Italy; vinai@iom.cnr.it (G.V.); capaldo@iom.cnr.it (P.C.); dalzilio@iom.cnr.it (S.D.Z.); piero.torelli@elettra.eu (P.T.); 2 Istituto Nanoscienze-CNR, Via Campi 213/a, 41125 Modena, Italy; riccardo.magrinmaffei@unimore.it (R.M.M.); alessandro.dibona@nano.cnr.it (A.d.B.); guido.paolicelli@nano.cnr.it (G.P.); andrea.mescola@nano.cnr.it (A.M.); sergio.daddato@unimore.it (S.D.); 3 Dipartimento di Scienze Fisiche Informatiche Matematiche, Università di Modena e Reggio Emilia, Via Campi 213/a, 41125 Modena, Italy; 4 A.P.E. Research srl, Area Science Park, Basovizza, ss14 Km 163.5, 34149 Trieste, Italy; matteo.locicero@aperesearch.com (M.L.C.); stefano.prato@aperesearch.com (S.P.); 5 Dipartimento di Fisica e Astronomia, Università di Padova, Via F Marzolo 8, 35131 Padova, Italy

**Keywords:** surface roughness, piezoelectricity, crystallinity, magnetron sputtering, metal electrodes, ZnO, thin films

## Abstract

The piezoelectric response of ZnO thin films in heterostructure-based devices is strictly related to their structure and morphology. We optimize the fabrication of piezoelectric ZnO to reduce its surface roughness, improving the crystalline quality, taking into consideration the role of the metal electrode underneath. The role of thermal treatments, as well as sputtering gas composition, is investigated by means of atomic force microscopy and x-ray diffraction. The results show an optimal reduction in surface roughness and at the same time a good crystalline quality when 75% O_2_ is introduced in the sputtering gas and deposition is performed between room temperature and 573 K. Subsequent annealing at 773 K further improves the film quality. The introduction of Ti or Pt as bottom electrode maintains a good surface and crystalline quality. By means of piezoelectric force microscope, we prove a piezoelectric response of the film in accordance with the literature, in spite of the low ZnO thickness and the reduced grain size, with a unipolar orientation and homogenous displacement when deposited on Ti electrode.

## 1. Introduction

Zinc oxide (ZnO) is extensively studied and employed because of its many applications, e.g., in sensors, photovoltaics or in piezoelectric devices [1,2]. Ceramic materials exhibit a macroscopic piezoelectric response when its crystalline structure has an axis that lacks inversion symmetry, with dipole moments aligned along the same direction. In the case of ZnO, this corresponds to the [0001] c-axis direction. ZnO has the advantage that it can be easily grown along its c-axis and in the form of a variety of nanostructures [3,4]. This fact has pushed the interest for this material in the field of sensors [5,6,7], actuators [8,9], and nanogenerators [10,11], in spite of the fact that the d_33_ coefficient is still about one order of magnitude lower than that of other common piezoelectric ceramic compounds [12]. In these applications, the typical ZnO film thickness is in the range of micrometers, with a maximum reported piezo response of tens of pm/V for an oriented or composite film [13,14,15], and up to 240 pm/V for V-doped ZnO [16,17]. Reducing the thickness, the piezoelectric response decreases, due to the reduction in grain size and crystal quality, making more difficult the exploitation of ZnO advantages in nanofabrication and nanodevices, where thinner oxide films are required [18,19]. Several deposition methods have been exploited to obtain piezoelectric ZnO [3], such as the hydrothermal treatment [20], the sol-gel spin coating technique [21], the ultrasonic spray pyrolysis technique [22] or physical vapor deposition (PVD) methods, such as magnetron sputtering [23,24], pulsed laser deposition [25,26] or laser molecular beam epitaxy [27]. Among them, PVD, and in particular magnetron sputter deposition, allow us to maintain a good control on the film growth conditions to obtain reproducible and controllable properties in c-axis-oriented ZnO films of tens to hundreds nm thickness [3]. As an example, sputter deposition allows obtaining good insulating films with controlled defects [28,29]. However, for sputter-deposited ZnO films, the typical d_33_ values reported in the literature are of few pm/V [30,31], lower than those obtained in films grown with other techniques. Values are particularly low when radio frequency (RF) is employed, due to generally smaller grains [32,33,34,35]. These small grains can reduce the effective displacement if their polarizations are not fully aligned, and the presence of impurities at grain boundaries can generate a large number of free electrons inside their structure, reducing the final resistivity. These free electrons move and screen the piezoelectric potential generated under mechanical actions, in turn reducing drastically its amplitude [36]. In spite of this, RF power has the advantage of producing stoichiometric films with a reduced roughness due to a well-controlled deposition rate [34]. This characteristic is important when the film has to be employed in heterostructures with an ultrathin top electrode, especially to improve the interface coupling properties [19,37,38]. To reach such a control over ZnO quality during sputter deposition, it is typically necessary to tune the growth parameters, such as gas pressure and mixture (e.g., with O_2_), target power density and deposition rate, as well as thermal treatments. Previous investigations have shown that the use of a high percentage of O_2_ in the gas mixture reduces the surface roughness in ZnO films [39,40]. An increased deposition temperature further improves the crystalline alignment along the c-axis, but it tends to increase surface roughness [41,42]. In addition, it is known that the resulting ZnO film quality, both roughness and c-axis orientation, strongly depends on the metal underneath [30,43,44]. In previous studies, several different metal films were used as bottom electrodes below the oxide film; however, with no clear optimization process [30,39,40,41,42,45,46]. The correct balance between these factors strongly determines the resulting piezoelectric response. While grain alignment along the [0001] c-axis and the control on polarity distribution (Zn- or O-termination of the grains) are decisive to improve piezoelectric response [41,45,46], the low surface roughness determines from one side the final flatness of the exposed sample surface, and on the other side a reduction in piezo coefficient [47]. 

From this scenario, it is therefore important to investigate the oxide properties at reduced thickness, by following and controlling its structural and morphological evolution, together with the influence of the substrate or seed material, namely the bottom metal electrode. 

In this work, we studied the combination of different growth parameters in sputter deposition, exploring the consequences on ZnO film quality in terms of roughness and crystal quality, also taking the effect of the substrate/bottom seed layer into consideration. We observed that the best condition is the use of 75% O_2_ in the sputtering gas, together with a variable deposition temperature between room temperature (RT) and 573 K. Further annealing at 773 K improves the film quality. When we apply this procedure on metal electrodes, the best results are obtained on Ti and Pt films, inducing a good piezoelectric response, more homogeneous in the vertical direction and unipolar in the case of the Ti electrode. 

## 2. Materials and Methods

ZnO films were deposited by RF magnetron sputtering from a ZnO 3” target (ZnO purity 99.99%) about 15 cm from the substrate in 3 × 10^−6^ mbar base pressure. To the oxide target was applied 120 W RF power, corresponding to a final rate between 0.04 and 0.1 nm.s^−1^, depending on the gas mixture. Gas pressure of 5 mTorr was obtained with a mixture of Ar and O_2_, with oxygen content varying from 0 to 75%, as described in the following section (flux 0–15 sccm). Film thickness was kept constant at 300 nm and was calibrated during the deposition by a quartz microbalance and checked after the growth by means of a profilometer. Deposition temperature was varied by resistive heating and measured with a calibrated thermocouple on the sample stage. Post-growth treatment was performed in air in a quartz tube furnace up to 973 K in a mixture of 50% N_2_ and 50% O_2_ flux. Substrates used for ZnO investigation (AFM, XRD) were n-doped Si(100) wafers covered by 680 nm thermal amorphous SiO_2_. The substrates were cleaned with acetone and isopropanol in an ultrasonic bath and dried with nitrogen. 

Then, the established procedure was used to grow ZnO on several metal electrodes (Ti, Pt, Al, Al/Cr and Pt/Cr). The thin metal films used as bottom electrodes were grown on MgO (001) substrates, after acetone and ethanol cleaning, at room temperature by means of electron beam evaporators in an atmosphere of around 5 × 10^−6^ mbar and a deposition rate of 0.01 nm.s^−1^, with a total thickness of 25 nm. Among the different metals used, here, we focus our study on the most promising ones, i.e., Pt and Ti. Similar measurements were, however, conducted on the other metals, and the results are shown in the Supplementary Material. The electrical resistance of the metal films was tested with a two-point probe after all preparation steps to check that sufficient electrical conductivity was left after the ZnO growth and the post-deposition treatments.

The morphology was investigated by means of an ambient pressure NTEGRA AURA NT-MDT Atomic Force Microscope (AFM) in semi-contact (tapping) mode with Si cantilevers. The crystallographic structure was characterized by X-Ray Diffraction (XRD), acquiring specular scans in θ−2θ geometry with a PanAnalytical X’Pert Pro diffractometer (Cu-Kα wavelength). Piezo force microscopy (PFM) measurements were performed with an A.P.E. Research instrument (model A100-AFM Plus version) in contact mode. We used AppNano doped diamond DD-ACTA tips, with an applied force of 10 nN, a 4 V DC bias and a ±3 V AC bias with a 10 kHz frequency.

## 3. Results

The film piezoelectric response strictly depends on its surface morphology and crystalline order. We thus investigated the most important parameters of film deposition on a SiO_2_/Si support for a 300 nm-thick ZnO film in order to obtain the best compromise. 

### 3.1. Temperature Optimization

**Figure 1:**
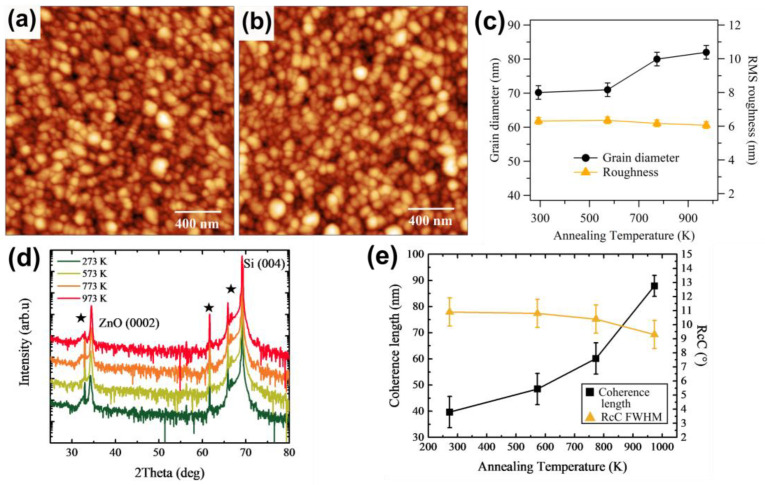
AFM images of size 2 × 2 µm^2^ (tapping mode) of (**a**) RT-grown ZnO film and (**b**) after post-growth annealing in 50% O_2_ and 50% N_2_ at 773 K. (**c**) Average grain diameter (black dot) and RMS roughness (orange triangle) as obtained by grain analysis of AFM images of surface shown in (**a**) as a function of annealing temperature. (**d**) XRD plots, (**e**) coherence length (black squares) and rocking curve FWHM (orange triangle) for increasing annealing temperature. The XRD signals are vertically shifted by a constant to better observe the differences. Stars in (**d**) indicate the replica peaks due to the non-monochromatized X-ray source.

Figure 1a shows the AFM image of the surface of ZnO film deposited at room temperature. To investigate the effect of temperature alone, we introduced only Ar in the plasma during sputter deposition. The surface was composed of grains with an average lateral diameter of 70 nm and an RMS roughness of 6.3 nm (Figure 1c). In XRD plots, only the ZnO(0002) peak is visible, together with the substrate diffraction signal (Figure 1d). This confirms the well-oriented growth of ZnO along the c-axis, as reported in the literature [3,24,48]. The position of the (0002) peak is shifted to a smaller angle (34.21°) than the expected bulk value of 34.55° [49]. This indicates an elongation along the c-axis of the wurtzite structure of the oxide (and typically an in-plane contraction due to elastic strain). The presence of amorphous silica on the substrate surface excludes an epitaxial origin of the compression. The same compression was assigned in previous works to the presence of defects in the oxide [50,51]. We previously demonstrated that our ZnO films grown at RT in Ar are over-stoichiometric in oxygen, due to the presence of Zn vacancies and O interstitials [29].

Further indications of the crystalline quality of the film are the 2θ (0002) and the rocking curve (RcC) full width at half maximum (FWHM) across the Bragg diffraction peak. The first is correlated with the coherence length by the Scherrer formula [52], representative of the size of the ordered crystalline domains along the axis perpendicular to the surface, while the RcC FWHM points out the grain alignment. Both values are reported in Figure 1e as a function of annealing temperature and are consistent with those reported in the literature [50,53].

When the film is annealed in a mixture of 50% O_2_ and 50% N_2_ after the deposition, the surface changes, as shown in Figure 1b. The grain size progressively increases and the roughness slightly reduces (Figure 1c). However, the improvement is modest. Additionally, when increasing the annealing temperature, the position of the (0002) peak shifts towards the bulk value (reaching 34.47° at 973 K) and becomes narrower, while the FWHM of the rocking curve also improves (Figure 1e). The structural improvement is a hint of the removal of defects (most probably O interstitials) and of an increased coherence length and grain alignment (thus, smaller RcCs), consistent with the grain size enlargement. Therefore, the annealing temperature is effective in reducing defectivity and improving long range order. The domain size extracted from XRD measurements starts at RT from much lower values than those measured with AFM (Figure 1e). We attribute this difference to the smaller size of the crystalline domains inside the grains. However, when the film is post-annealed up to 973 K, the crystalline domains and the grains almost coincide (Figure 1e and Figure 1c, respectively), indicating a strong ordering inside the grains and the removal of internal grain boundaries, as confirmed by the reduction in RcC FWHM. Small discrepancies can be related to the non-spherical shape of the grains, which have been often observed to be elongated along the c-axis [3,39,54,55].

We therefore identify 773 K as the best compromise for post-growth annealing temperature to obtain surface quality improvements and being at the same time compatible with deposition on most metal electrodes that can be used in piezoelectric devices.

**Figure 2:**
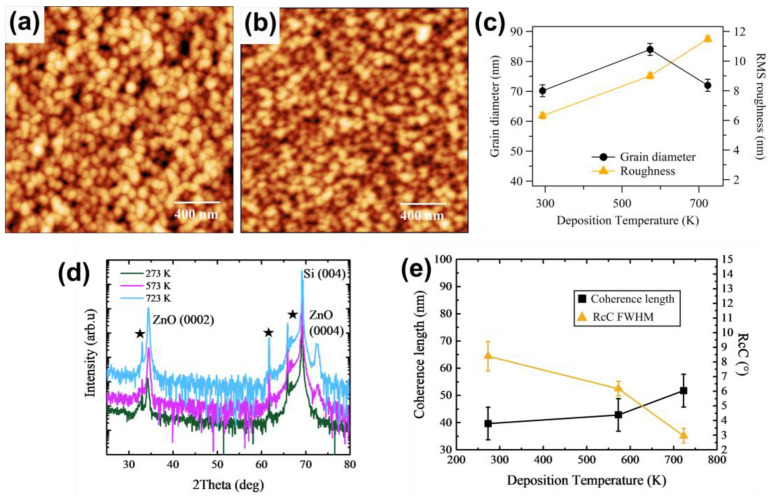
AFM images of size 2 × 2 µm^2^ of 300 nm ZnO deposited at (**a**) 573 K and (**b**) 723 K in 100% Ar. (**c**) Average grain diameter (black dot) and RMS roughness (orange triangle) as obtained by grain analysis of AFM images of surfaces as a function of deposition temperature. (**d**) XRD plots, (**e**) coherence length (black squares) and rocking curve FWHM (orange triangles) for increasing deposition temperature. The XRD signals are vertically shifted to better observe the peaks.

When the deposition temperature is increased to 573 K, AFM image shows grains that increase in diameter to about 85 nm (Figure 2a), then reduce at 723 K (Figure 2b), followed by a continuous increase in roughness (Figure 2c). Compared to the results on surfaces after post-growth annealing (Figure 1c), here, we observe the formation of faceted grains that have a less smooth shape. At the same time, the (0002) peak is more intense, revealing the second-order ZnO (0004) diffraction peak (Figure 2d), and is closer to the bulk relaxed position (34.41° at 723 K), while the rocking curve dramatically shrinks (Figure 2e). This behavior is compatible with a reduced defect density and a better crystalline quality, improving with increasing temperature, related to the modifications of the grain size and shape, even if in this case the crystalline domains remain smaller than the grain size.

Despite clear improvements in the crystalline quality with annealing treatments—either post- or during growth—the surface roughness does not follow the same trend. This is consistent with previous works [41,42]; however, the RMS roughness has to be further reduced to be compatible with a heterostructure-based device implementation including an ultrathin top metal electrode.

### 3.2. Role of Oxygen in Sputtering Gas

To accomplish this goal, we studied the use of oxygen introduced in the sputtering gas [39], combined with the best temperature conditions found before. The presence of oxygen in the sputtering plasma is expected to reduce the deposition rate and favor the growth with a lower final roughness. However, it can increase the already abundant presence of oxygen in the film, therefore increasing the density of O interstitials. Furthermore, it will also increase the electrical resistivity of the ZnO film [56,57], even if the films presented here are already insulating also in the absence of oxygen, probably due to the O defects that compensate for the presence of native H donors [28].

**Figure 3:**
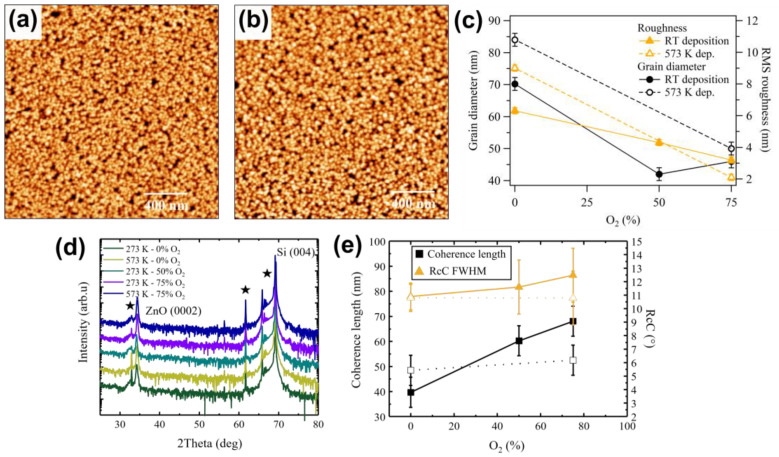
AFM images of size 2 × 2 µm^2^ of ZnO deposited with (**a**) 50% and (**b**) 75% O_2_ at RT. (**c**) Average grain diameter (black dot) and RMS roughness (orange triangle) as obtained by grain analysis of AFM images of surface as a function of oxygen content during deposition. Empty markers are the corresponding curves obtained from films deposited at 573 K. (**d**) XRD plots, (**e**) coherence length (black squares) and rocking curve FWHM (orange triangles) for increasing oxygen content for RT (fill triangles and squares) and 573 K depositions (empty triangles and squares). The XRD signals are vertically shifted by a constant.

When 50% O_2_ is added to the sputtering gas, the surface maintains a granular morphology, as shown in Figure 3a, but the surface roughness decreases markedly from 6.3 to 4.3 nm, while the grain diameter decreases from 70 down to 42 nm (Figure 3c). Both roughness and grain size remain low when further increasing the oxygen content to 75% (Figure 3b). The (0002) 2θ peak shifts from 34.21° to lower Bragg angle (34.19°, Figure 3d), with a slightly broader rocking curve FWHM (Figure 3e). As expected, the inclusion of more O interstitials leads to a higher deformation in the film. Finally, the coherence length increases and becomes higher than the grain size obtained from AFM measurements (Figure 3e). This can be assigned to the formation of grains elongated in the c-axis direction [3,39,54,55], with a reduced degree of in-plane ordering. Since the piezoelectric response of the film is expected in the direction normal to the surface, i.e., [0001], we choose 75% O_2_ as the most suitable for the device application. If we then combine the growth in 75% oxygen and a higher deposition temperature (573 K—Figure 3c), roughness further decreases, giving a final value of 2.1 nm, while at the same time the mean grain size remains almost unchanged. On the other hand, the coherence length rises up to 68 nm, keeping a relatively unchanged RcC (Figure 3e).

To further improve roughness and crystallinity, we performed the post-growth annealing at 773 K identified in Section 3.1. In this case, the improvement in terms of surface roughness is modest for the film grown at RT (from 3.1 to 2.6 nm) and negligible for the 573 K film (not shown). Additionally, the XRD shows a significant decrease in the RcC FWHM, keeping a modest coherence length.

We can here conclude that the best procedure to grow optimized 300 nm ZnO films on SiO_2_/Si happens through deposition by reactive sputtering with 75% O_2_ (either at RT or 573 K), followed by an annealing at 773 K.

### 3.3. Deposition on Metal Electrodes 

Finally, the choice of the substrate is fundamental for determining the properties of the sputtered film. If we then apply the best identified procedures for a SiO_2_/Si substrate on metal electrodes, the results are not trivial.

We chose several metal electrode films to be tested: Ti, Pt, Al, Al/Cr and Pt/Cr, all grown on MgO(001), with the metal thicknesses set to 25 nm. The reduced metal thickness is motivated by the necessity to fabricate compact, low-cost devices and possibly patterned bottom electrodes such as those in [19]. Then, we checked by AFM and XRD the final quality of the obtained ZnO films. Despite similar deposition conditions, the results are extremely different. Grain size and roughness are significantly different as a function of the chosen metal, both being quite high for all metals except Ti and Pt. Consistently, the crystalline order is poor for all metal electrodes excluding Pt and Ti, with the (0002) Bragg peak sometimes hardly visible. The full data and AFM images are reported in the Supplementary Material. We show in Table 1 the values of surface quality and crystallinity for Ti and Pt electrodes. For Ti and Pt, in contrast with the other metal supports, the surface roughness is low and the crystalline order is high, especially on Ti. Metallic Ti grows hexagonally on MgO(001) [58], while Pt tends to grow in (111) orientation at room temperature on MgO (001) [59]. Both metals thus favor ZnO(0001) growth with c-axis orientation, with Ti favoring a better order locally due to the smaller lattice mismatch (10.1%) with respect to Pt (17.5%). Other metals have a more favorable matching with MgO(001) in a square epitaxy, and so a good ordering of zinc oxide is less favored.

As already observed on Si substrates, in these cases the ZnO thin films are also strained at RT due to the presence of defects, but become relaxed when the deposition temperature is rising. The thermal treatment increases the quality of the films, reducing the FWHM of the RcC and increasing the coherence length, along with keeping a low surface roughness. After the post-growth annealing, a coherent length up to a hundred nm (better than in the case of SiO_2_/Si substrate) is found in most of the measured cases. The best structural and morphological properties are found on the Ti electrode. These points highlight the importance of the seed layer, and that a compromise should be found.

### 3.4. Piezoelectric Properties

**Figure 4:**
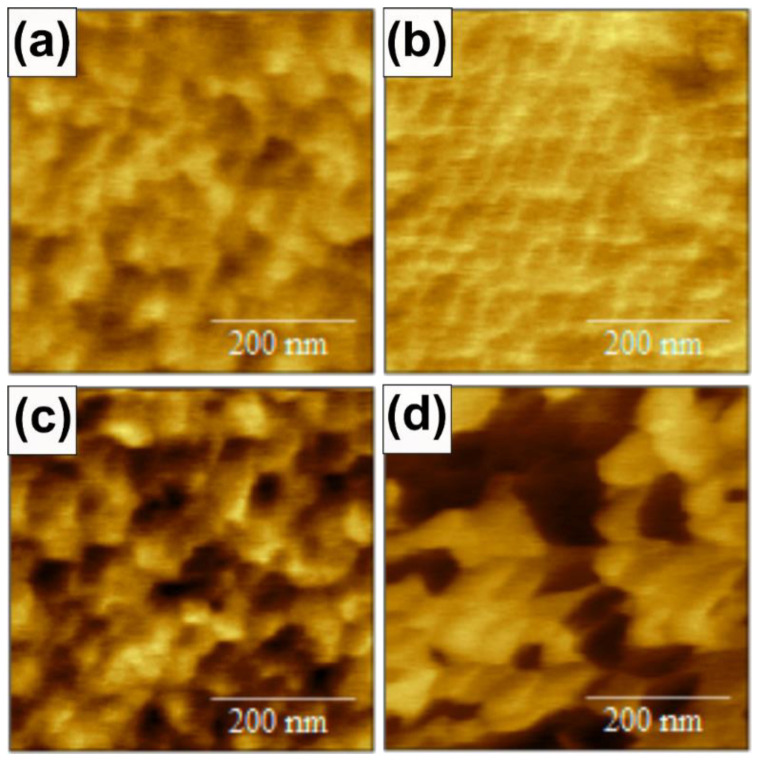
PFM images of size 0.5 × 0.5 µm^2^ of ZnO deposited with 75% O_2_ at RT (**a**,**c**) and at 573 K (**b**,**d**) on Ti (**a**,**b**) and on Pt (**c**,**d**). The contrast represents the effective piezoelectric response for an applied alternating voltage of ±3 V over a continuous bias voltage of 4 V.

To conclude our investigation of the characterization of the film for device applications, we measured the piezoelectric response by means of PFM on the ZnO films grown at RT and 573 K on Pt and Ti bottom electrodes. A sketch of the PFM measurement configuration is shown in the Supplementary Material. The resulting PFM images are shown in Figure 4 for the different studied cases: ZnO/Ti deposited at RT (a) and at 573K (b), and ZnO/Pt deposited at RT (c) and at 573K (d). The images show the effective piezoelectric response extracted from topography, PFM displacement and phase responses (see Figure S7) when an alternating voltage of ± 3 V over a continuous 4 V voltage is applied between the tip, acting as a top electrode, and the bottom metal electrode. We choose 4 ± 3 V to remain below the breakdown voltage expected for ZnO thin films [60]. No initial applied bias was used to polarize the samples. For [0001]-oriented ZnO, this response should be dominated by the vertical displacement over the in-plane piezo coefficients. 

Figure 4 highlights drastically different contrasts on the different ZnO surfaces. As observed in [61], different ZnO grains present different piezo properties, which could be detrimental for device applications. In the images, we can observe that ZnO on Ti deposited at 573 K has a quite homogeneous response (Figure 4b). On the contrary, on Pt deposited at 573 K, opposite contrasts are observed when passing from one grain to another, corresponding to fully opposite phases (Figure 4d). The images indicate the presence of only two orientations, assigned to (0001) and $\left(000\bar{1}\right)$ polarity, as evidenced also in XRD. Similar observations with much reduced contrast can be made for the two other cases deposited at RT (Figure 4a,c). As a consequence, we can state that on Ti electrodes the ZnO film has a unique polarity, as highly desirable for piezo applications [41,45,46].

To carefully evaluate the full piezo response, in-plane PFM was also tested, where no signal was observed (not shown here). Considering that we have essentially vertical displacement, we obtain 1.5 ± 0.3 and 1.9 ± 0.3 pm·V^−1^ for deposition on Ti at RT and 573 K, 1.4 ± 0.3 and approx. 1.5 ± 0.3 pm·V^−1^ for deposition on Pt at RT and 573 K, respectively. Despite no initial bias used to fully polarize the samples, these values are comparable with those reported in the literature for ZnO films obtained by RF sputter deposition [30,31,32,33,34,35]. 

## 4. Conclusions

We studied the combination of sputtering gas composition and temperature treatments during the fabrication of 300 nm-thick ZnO films to obtain the best film quality in terms of surface roughness and crystal quality. We observed that the best condition is the use of 75% O_2_ in the sputtering gas, together with a variable deposition temperature between RT and 573 K. In these conditions, the film has a surface roughness around 2–3 nm when deposited on SiO_2_/Si, a good orientation of the film along the c-axis and columnar grains of about 45 nm diameter in the surface plane. Some degree of disorder is due to the presence of defects, most probably O interstitials. When we apply this procedure for the growth of ZnO on different metal bottom electrodes, the best results are obtained in the case of Pt and Ti films, inducing a moderate piezoelectric response, with only vertical displacement and a homogeneous polarity orientation in the case of Ti. On other metals the growth is completely different, with high roughness and a poor crystalline quality.

## Figures and Tables

**Table 1:** Measured experimental parameters of ZnO films on two different electrodes, Pt and Ti, deposited in 75% O_2_ at RT and 573 K, and then annealed at 773 K in 50% O_2_ and 50% N_2_ atmosphere.

Bottom Electrode	Deposition Conditions	RMS Roughness (nm)	Grain Diameter (nm)	Bragg Peak (°)	Coherence Length (nm)	RcC of the (0002) Bragg Peak (°)
Pt	273 K	3.4 ± 0.5	49 ± 3	34.21 ± 0.02	30 ± 4	>20
	Annealed 773 K	3.5 ± 0.3	54 ± 3	34.34 ± 0.02	42 ± 3	>20
	573 K	6.0 ± 0.2	55 ± 5	34.31 ± 0.02	33 ± 4	>20
	Annealed 773 K	4.5 ± 0.4	70 ± 5	34.39 ± 0.02	104 ± 2	>20
Ti	273 K	2.3 ± 0.3	39 ± 4	34.28 ± 0.02	39 ± 3	16 ± 2
	Annealed 773 K	15.2 ± 1	130 ± 8	34.37 ± 0.02	102 ± 2	7 ± 2
	573 K	3.4 ± 0.2	43 ± 3	34.33 ± 0.02	80 ± 2	3 ± 1
	Annealed 773 K	2.3 ± 0.3	44 ± 2	34.39 ± 0.02	115 ± 2	3 ± 1

## Data Availability

The data that support the findings of this study are available from the corresponding authors upon reasonable request.

## Supplementary Materials

The following images/table are available online at https://www.mdpi.com/article/10.3390/s21186114/s1, Figure S1: AFM of 300 nm ZnO grown on Ti and Pt on MgO(001) at RT and 573 K; Figure S2: XRD θ−2θ scans on ZnO film on Pt and Ti on MgO(001); Figure S3: AFM images of ZnO grown on Pt and Ti after post-growth annealing at 773 K; Figure S4: AFM images of ZnO grown other metal electrodes; Figure S5: XRD θ−2θ scans on ZnO on other metal electrodes on MgO(001); Figure S6: Sketch of PFM and film heterostructure; Figure S7: Topography, phase and amplitude measured in PFM on ZnO films grown on Pt and Ti at RT and 573 K; Table S1: Measured experimental parameters from AFM images of ZnO films on the different electrodes, deposited in 75% O_2_ at RT and 573 K.
