# Supplementary material for: ZnO Thin Films Growth Optimization for Piezoelectric Application
Source: Sensors (Basel). 2021 Sep 12;21(18):6114. doi: 10.3390/s21186114 (PMC8472809; doi:10.3390/s21186114)
Supplement: Supplementary file 1 [file sensors-21-06114-s001.zip › sensors-1342962-supplementary.pdf]

# Supplementary Material

# ZnO Thin Films Growth Optimization for Piezoelectric Application

V. Polewczyk [1, *], R. Magrin Maffei [2,3], G. Vinai [1], M. Lo Cicero [4], S. Prato [4], P. Capaldo [1,5], S. Dal Zilio [1], A. di Bona [2], G. Paolicelli [2], A. Mescola [2], S. D'Addato [2,3], P. Torelli [1] and S. Benedetti [2,*]

[1] *Istituto Officina dei Materiali (IOM)-CNR, Laboratorio TASC, 34149 Trieste, Italy*

[2] *Istituto Nanoscienze-CNR, via Campi 213/a, 41125 Modena, Italy*

[3] *Dipartimento di Scienze Fisiche Informatiche Matematiche, Università di Modena e Reggio Emilia, via Campi 213/a, 41125 Modena, Italy*

[4] A.P.E. Research srl, Area Science Park, Basovizza, ss14 Km 163.5, I-34149, Trieste, Italy

[5] *Dipartimento di Fisica e Astronomia, Università di Padova,* via F Marzolo 8, I-35131, Padova, Italy

* stefania.benedetti@nano.cnr.it

* polewczyk@iom.cnr.it

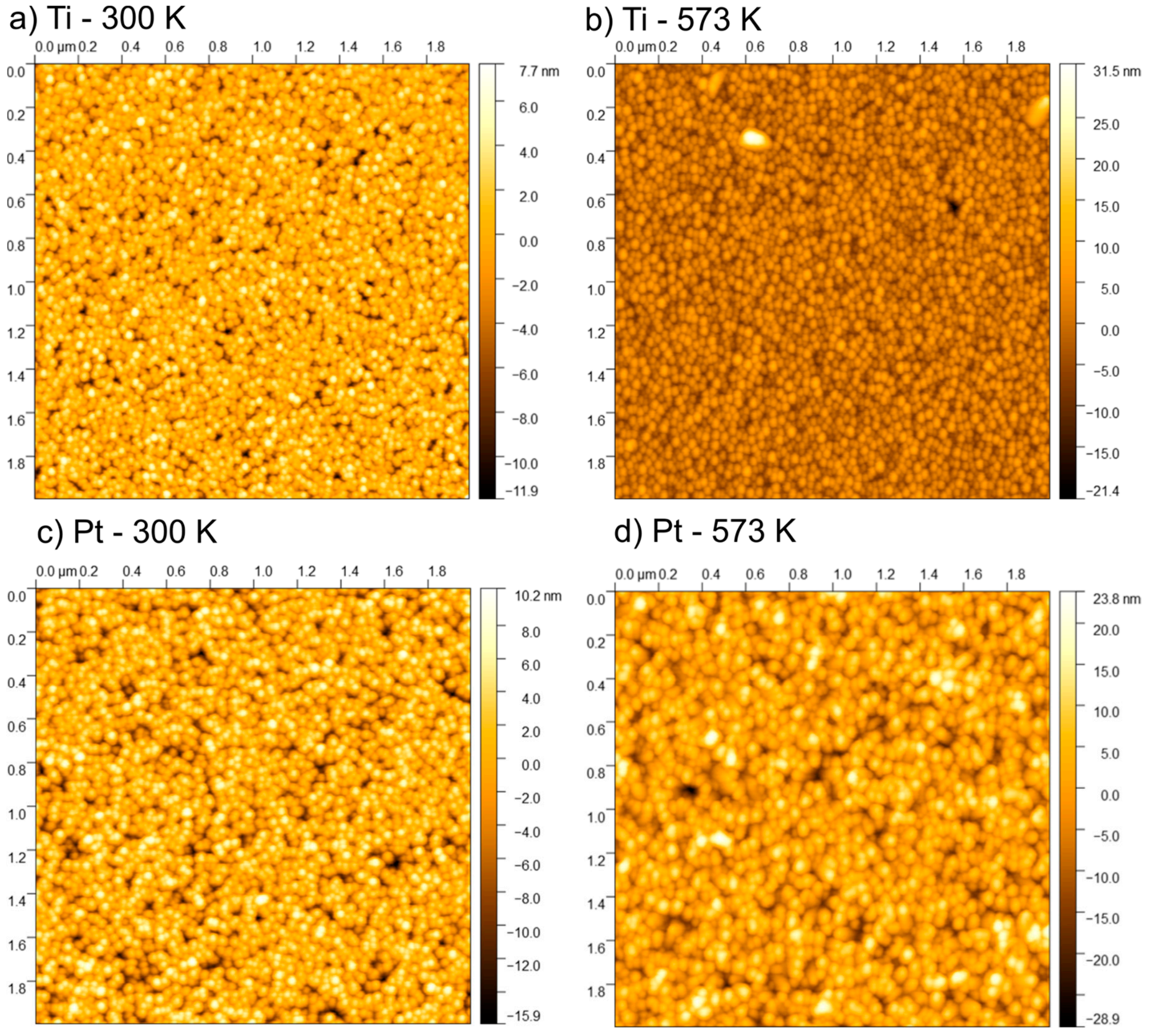


**Figure S1.** AFM 2x2 μm$^2$ images of 300 nm ZnO grown on a,b) 25 nm Ti and c,d) 25 nm Pt on MgO(001) at (a,c) RT and (b,d) 573 K.

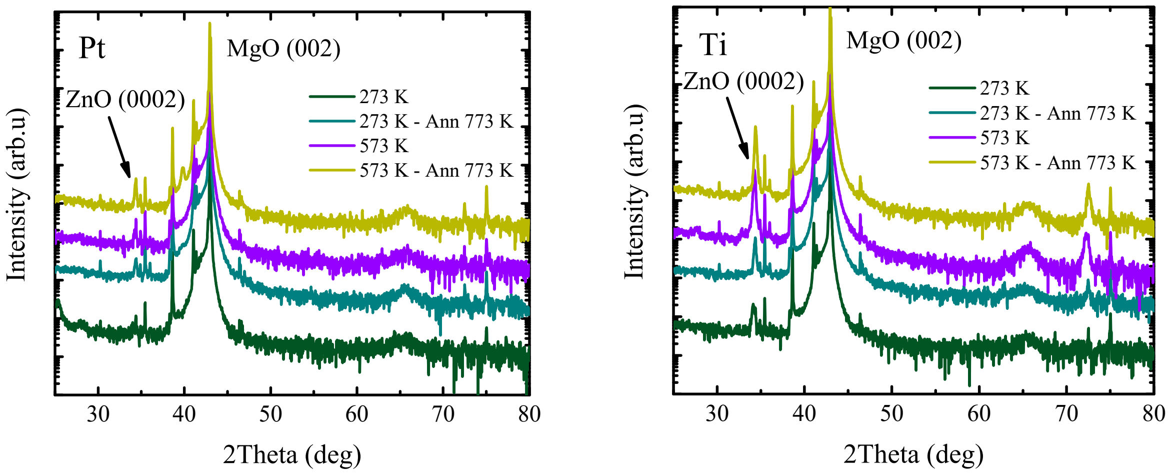


**Figure S2.** XRD $\theta - 2\theta$ scans on 300 nm ZnO film on 25 nm Pt (left) and 25 nm Ti (right) on MgO(001).

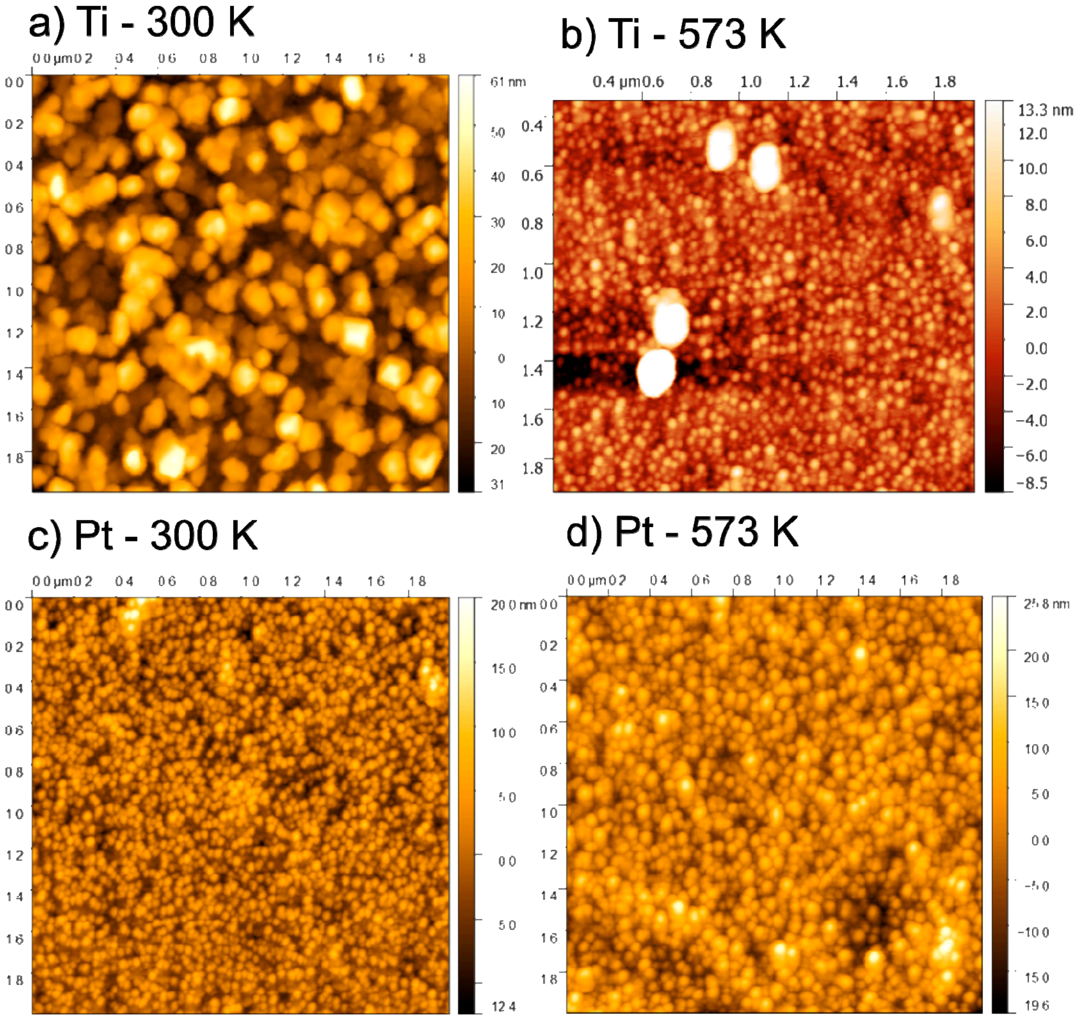


**Figure S3.** AFM 2x2 $\mu m^2$ images of 300 nm ZnO grown on electrodes as in Figure S1 after post-growth annealing at 773 K in 50% $O_2$ and 50% $N_2$.

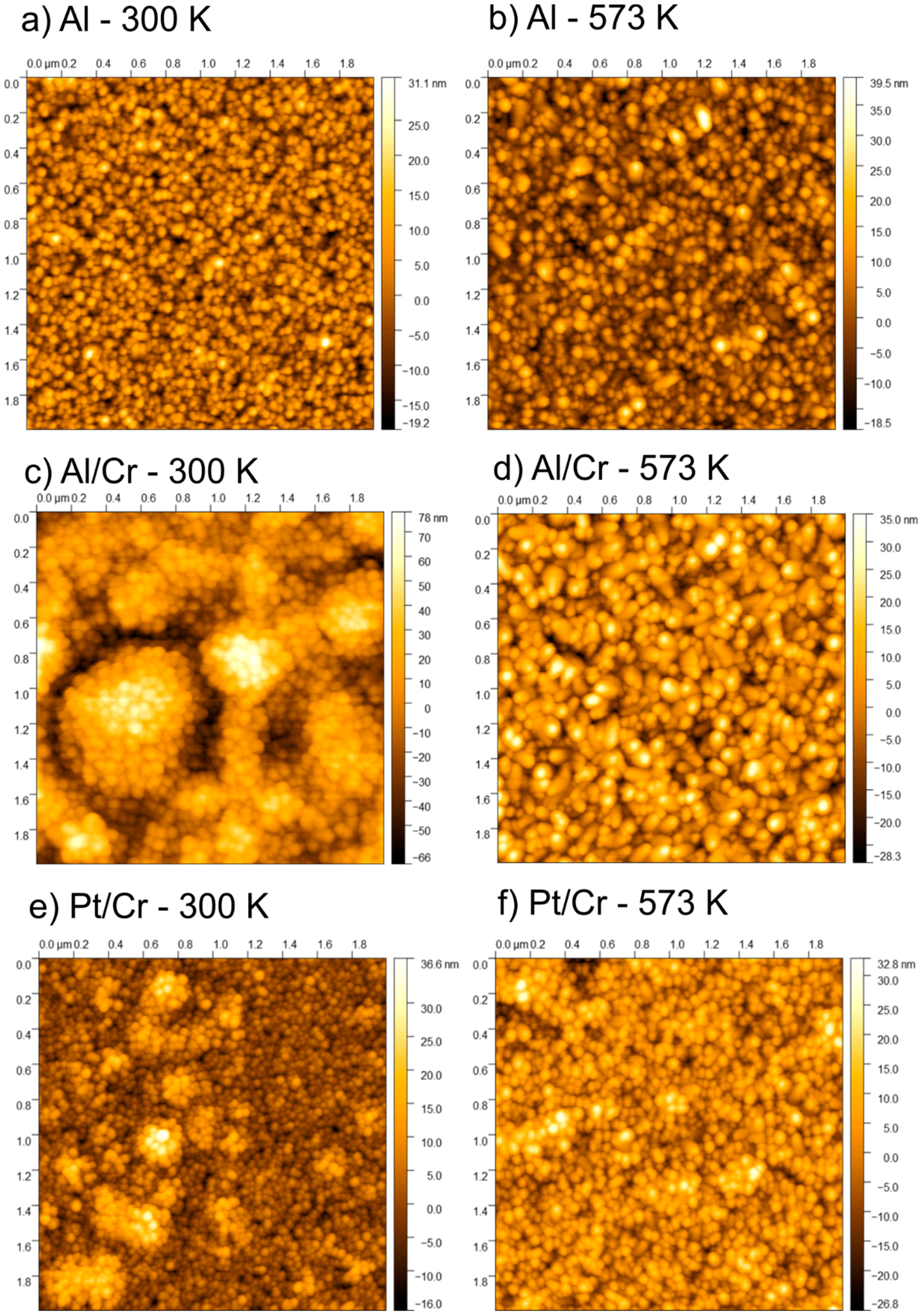


**Figure S4.** AFM 2x2 µm$^2$ images of 300 nm ZnO grown on a,b) 25 nm Al, c,d) 20 nm Al/5 nm Cr, e,f) 20 nm Pt/5 nm Cr on MgO(001) at (a,c,e) RT and (b,d,f) 573 K.

**Table S1.** Measured experimental parameters from AFM images of ZnO films on the different electrodes, deposited in 75% $O_2$ at RT and 573 K.

| Sample (ZnO deposition temperature) | RMS roughness (nm) | Grain diameter (nm) |
|---|---|---|
| Al (RT) | 13 ± 7 | 28 ± 2 |
| Al (573 K) | 6.7 ± 0.1 | 30 ± 4 |
| Ti (RT) | 2.3 ± 0.3 | 19 ± 2 |
| Ti (573 K) | 3.4 ± 0.2 | 21 ± 1 |
| Pt (RT) | 3.4 ± 0.5 | 24 ± 2 |
| Pt (573 K) | 6.0 ± 0.2 | 28 ± 3 |
| Al/Cr (RT) | 6.1 ± 0.2 | 27 ± 3 |
| Al/Cr (573 K) | 9.1 ± 0.6 | 34 ± 7 |
| Pt/Cr (RT) | 5.7 ± 0.8 | 22 ± 2 |
| Pt/Cr (573 K) | 7 ± 1 | 28 ± 3 |

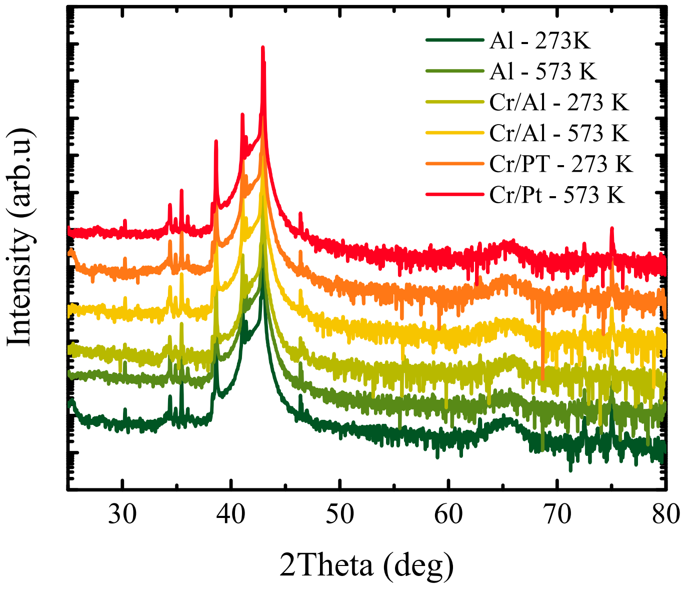


**Figure S5.** XRD $\theta - 2\theta$ scans on 300 nm ZnO film on Al, Al/Cr and Pt/Cr electrodes on MgO(001).

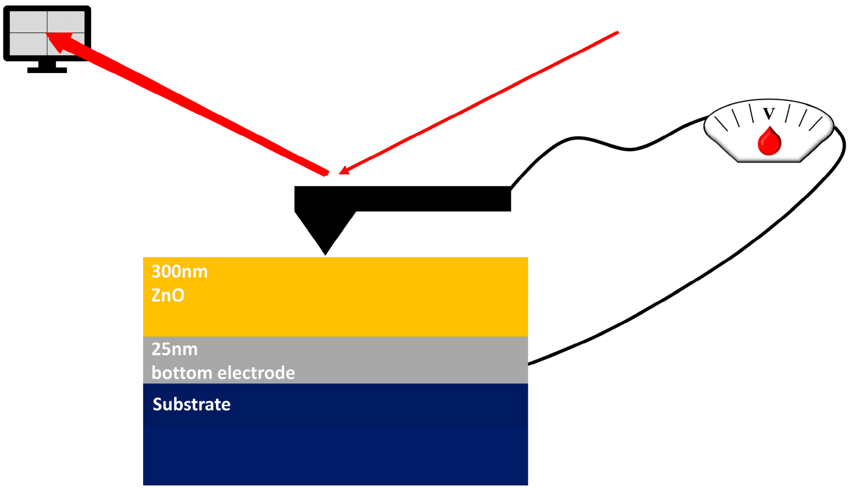


**Figure S6.** Sketch of PFM and film heterostructure.

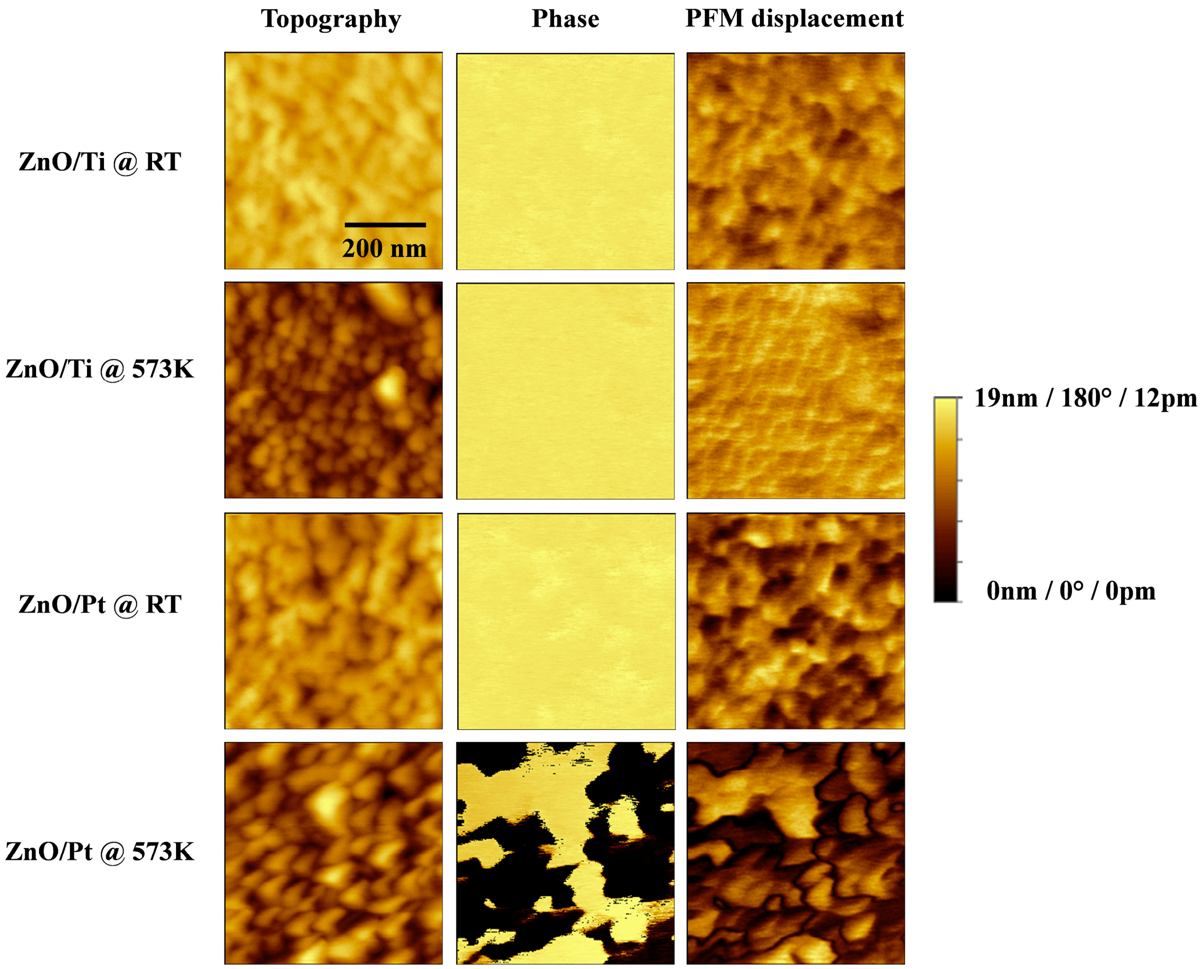


**Figure S7.** Topography, phase and amplitude measured in PFM on ZnO films grown on Pt and Ti at RT and 573 K using a common scale between the different cases.
